# Liver secretin receptor predicts portoenterostomy outcomes and liver injury in biliary atresia

**DOI:** 10.1038/s41598-022-11140-9

**Published:** 2022-05-04

**Authors:** Nimish Godbole, Iiris Nyholm, Maria Hukkinen, Joseph R. Davidson, Athanasios Tyraskis, Jouko Lohi, Päivi Heikkilä, Katja Eloranta, Marjut Pihlajoki, Mark Davenport, Markku Heikinheimo, Antti Kyrönlahti, Mikko P. Pakarinen

**Affiliations:** 1 Pediatric Research Center, Children’s Hospital, University of Helsinki and Helsinki University Hospital, Helsinki, Finland; 2 Section of Pediatric Surgery, Pediatric Liver and Gut Research Group and Pediatric Research Center, Children’s Hospital, University of Helsinki and Helsinki University Hospital, Children’s Hospital, P.O. Box 281, 00029 HUS Helsinki, Finland; 3 Department of Pediatric Surgery, GOS-UCL Institute of Child Health, London, UK; 4 Department of Pediatric Surgery, King’s College Hospital, London, UK; 5 Department of Pathology, University of Helsinki and Helsinki University Hospital, Helsinki, Finland; 6 Department of Pediatrics, Washington University in St. Louis, St. Louis, MO USA

**Keywords:** Biomarkers, Biliary tract disease, Paediatric research

## Abstract

Biliary atresia (BA) is a chronic neonatal cholangiopathy characterized by fibroinflammatory bile duct damage. Reliable biomarkers for predicting native liver survival (NLS) following portoenterostomy (PE) surgery are lacking. Herein we explore the utility of 22 preidentified profibrotic molecules closely connected to ductular reaction (DR) and prevailing after successful PE (SPE), in predicting PE outcomes and liver injury. We used qPCR and immunohistochemistry in a BA cohort including liver samples obtained at PE (n = 53) and during postoperative follow-up after SPE (n = 25). Of the 13 genes over-expressed in relation to cholestatic age-matched controls at PE, only secretin receptor (*SCTR*) expression predicted cumulative 5-year NLS and clearance of jaundice. Patients in the highest *SCTR* expression tertile showed 34–55% lower NLS than other groups at 1–5 years after PE (*P* = 0.006–0.04 for each year). *SCTR* expression was also significantly lower [42 (24–63) vs 75 (39–107) fold, *P* = 0.015] among those who normalized their serum bilirubin after PE. Liver SCTR expression localized in cholangiocytes and correlated positively with liver fibrosis, DR, and transcriptional markers of fibrosis (*ACTA2*) and cholangiocytes (*KRT7*, *KRT19*) both at PE and after SPE. SCTR is a promising prognostic marker for PE outcomes and associates with liver injury in BA.

## Introduction

Biliary atresia (BA) is a rare neonatal cholangiopathy characterized by progressive fibroinflammatory damage of the extra and intra hepatic bile ducts and obstruction of the bile flow leading to fatal biliary cirrhosis, if left untreated^1^. Current first line treatment for BA includes early diagnosis and surgical attempt to restore the bile flow with portoenterostomy (PE), also known as the Kasai procedure^1,2^. Several patients and treatment related factors may affect the success of PE surgery, including patient age at PE, stage of liver fibrosis, use of post-operative steroids as well as surgical expertise^2,3^. Even after successful PE (SPE) and initial normalization of serum bilirubin concentration, majority of patients will require liver transplantation (LT) before the age of 20 years due to ongoing progression of the chronic cholangiopathy and liver fibrosis^4^, making BA the commonest cause of LT in the pediatric population^1,2^.

Despite very active research during the last decades^5^, little progression has been made in improving native liver survival (NLS) in BA^1,4,6^. Normalization of serum bilirubin concentration after PE^7^ and AST-to-platelet ratio index (APRi)^8^ remain the best established clinical prognosticators for NLS in BA. However, there remains an unmet need for reliable and accurate biomarkers to predict outcomes of PE at presentation. Recently, liver gene expression signatures^9^ as well as transient elastography^10^ at the time of PE have shown initial promise in predicting PE outcomes. With emerging novel medical therapies for pediatric cholestatic disorders including BA^11,12^, understanding the pathophysiology of BA liver injury and how it relates to PE outcomes has become increasingly important^4^. As PE outcomes in individual patients remain highly variable, identification of accurate prognostic biomarkers reflecting the underlying key mechanisms driving liver injury would expedite targeted medical therapy, timing of LT and patient counseling^13,14^. In this respect, ductular reaction (DR), the histopathological hallmark of cholestatic disorders including BA, has been increasingly viewed as a potential therapeutic target for preventing progressive liver fibrosis^15^. Proliferating reactive cholangiocytes are of interest in this respect given that they are essential DR effector cells with the ability to activate active alpha smooth muscle actin (α-SMA) expressing myofibroblasts and fibrogenesis^15,16^.

Using mRNA sequencing, we have recently characterized an extracellular matrix (ECM) molecular fingerprint in BA, a set of 22 genes mechanistically connected to DR and biliary fibrosis^17^. These 22 genes, differentially overexpressed at the time of PE, sustained their highly increased BA specific expression one year after SPE, suggesting their mechanistic importance in driving BA liver injury. The aim of this study was to further explore the prognostic and pathophysiological significance of these 22 candidate genes by utilizing a large sample set and a long follow-up period extending to > 8 years after SPE. We hypothesized that by correlating the liver expression of these genes with the key outcome measures of PE as well as markers of liver fibrosis and DR, we could identify reliable prognostic markers with pathophysiological relevance to improve clinical management of BA.

## Results

### Patient characteristics

There were 75 patients included in the study who underwent PE at median age of 64 (42–77) days. 49 (65%) patients cleared their jaundice (bilirubin < 20 µmol/L) with 16 of them requiring subsequent LT. Additionally, 23 patients underwent LT who did not clear their jaundice. Overall 39 patients had LT at median age of 1.9 (0.9–2.5) years. NLS for all included patients was 80% (95% confidence interval (CI) 71–89), 66% (95% CI 56–78) and 47% (95% CI 36–60) at 1, 2 and 5 years, respectively. Detailed patient characteristics are presented in Table 1.

**Table 1:** Baseline patient characteristics.

	All patients (n = 75)	PE (n = 53)	Follow-up after successful PE (n = 25)
Age at PE, d	64 (42–77)	65 (44–75)	62 (37–79)
Follow-up after PE, y	3.0 (1.2–9.8)	1.9 (0.8–4.3)	10.1 (5.6–13.2)
**Type of BA, n (%)**			
1 or 2	2 (3)	0 (0)	2 (8)
3	73 (97)	53 (100)	23 (92)
Splenic malformation, n (%)	13 (17)	8 (15)	5 (20)
Cystic disease, n (%)	11 (15)	8 (15)	3 (12)
Clearance of Jaundice, n (%)	49 (65)	27 (51)	25 (100)
Liver transplantation, n (%)	39 (52)	33 (62)	6 (24)
Age at liver transplantation, y	1.9 (0.9–2.5)	1.4 (0.8–2.3)	8.1 (3.8–9.4)
Died without transplantation, n	2	2	0
**Liver Biochemistry**			
Bilirubin total, μmol/L	124 (13–156)	144 (133–184)	11 (7–17)
GGT (U/L)	251 (87–683)	535 (251–904)	59 (23–179)
AST (U/L)	163 (91–252)	209 (171–284)	78 (42–106)
ALT (U/L)	75 (37–176)	136 (87–367)	54 (27–91)
APRi	0.98 (0.58–1.69)	0.89 (0.59–1.35)	1.26 (0.57–1.92)

Data are median (IQR) or frequencies (%). Liver biopsies were obtained at portoenterostomy (PE) in 50 patients, during postoperative follow-up after successful PE in 22 patients, and both at PE and during follow-up in 3 patients.

### Expression of ECM molecular fingerprint genes in relation to controls

**Figure 1:**
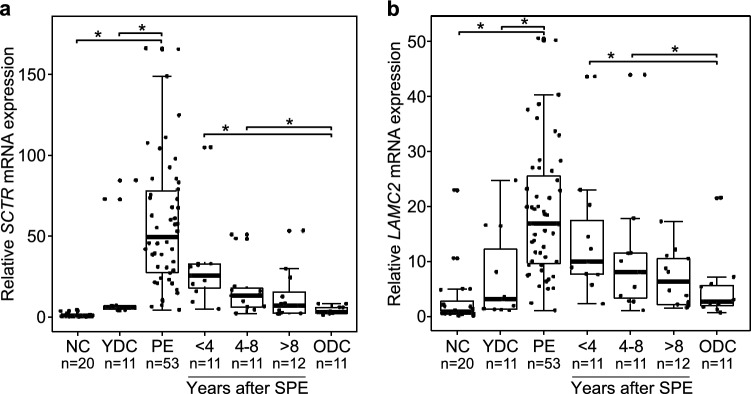
Relative liver mRNA expression of secretin receptor (*SCTR*) and laminin subunit gamma-2 (*LAMC2*). Box plots (median, interquartile range and 90th percentile with jittered data points) of liver (**a**) *SCTR* and (**b**) *LAMC2* expression in normal controls (NC), young disease controls (YDC), old disease controls (ODC), biliary atresia patients at portoenterostomy (PE) and during follow-up after successful PE (SPE). *P < 0.05.

As expected, liver expression of all 22 genes of the ECM molecular fingerprint were significantly overexpressed at the time of PE, when compared to normal controls. However, only 13 of these 22 genes showed significantly increased expression at PE also when compared to cholestatic YDC group, signifying BA specific overexpression (Fig. 1 and in Supplementary Fig. S1 available online). Of these 13 genes, only expression of *SCTR*, encoding Secretin receptor, and *LAMC2*, encoding Laminin subunit gamma-2, decreased progressively step by step with increasing NLS period after SPE reaching a comparable expression level with ODC group after 8 years (Fig. 1). The expression profiles for the 9 genes without BA specific overexpression are displayed in Supplementary Fig. S2 available online.

### SCTR expression at PE predicted native liver survival and clearance of jaundice

**Figure 2:**
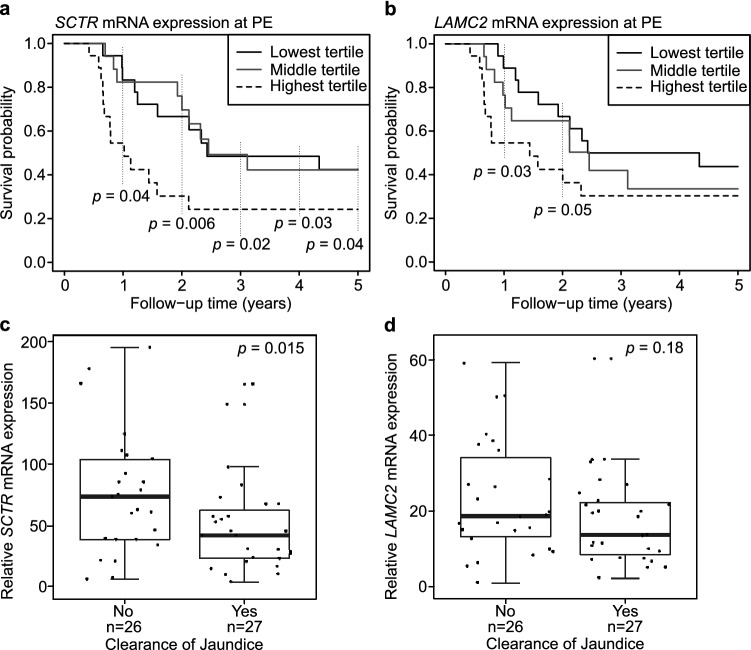
Portoenterostomy (PE) outcomes according to secretin receptor (*SCTR*) and laminin subunit gamma-2 (*LAMC2*) expression. Kaplan–Meier survival curves for native liver survival according to tertiles of mRNA liver expression in (**a**) *SCTR* and (**b**) *LAMC2* at PE (n = 53). Significant p-values are shown separately for each follow-up year. Expression of (**c**) *SCTR* and (**d**) *LAMC2* according to clearance of jaundice following PE.

Next, we evaluated the effect on NLS and clearance of jaundice following PE for all 22 genes. To analyze the effect of gene expression on NLS, we divided mRNA expression levels for each gene at PE into tertiles and found only two genes, *SCTR* and *LAMC2*, which significantly associated with NLS (Fig. 2). Notably, only *SCTR* expression predicted 5-year NLS so that patients in the highest *SCTR* expression tertile consistently showed significantly lower NLS than the other two groups by 34–55% at each year after PE. Furthermore, of all genes studied, only *SCTR* expression associated with clearance of jaundice along with *COL15A1* (Fig. 2). In tune with decreased NLS in the highest expression tertile, median *SCTR* mRNA expression level was significantly lower in patients, who normalized their serum bilirubin following PE than patients who did not [42 (24–63) vs 75 (39–107) fold, *P* = 0.015]. The respective figures for *COL15A1* were [12 (8.3–15) vs 19 (12–22) fold, *P* = 0.030]. Although *LAMC2* expression predicted 2-year NLS, it was not associated with clearance of jaundice (Fig. 2). Additionally, when dividing mRNA expression values into two halves by their medians, *SCTR* maintained its significant prognostic potential for the first two years after PE while *LAMC2* lost its predictive value (Supplementary Fig. S3 available online). Kaplan–Meier NLS curves for other genes are presented in Supplementary Fig. S4 and Supplementary Fig. S5 available online, according to their overexpression specificity for BA as described above.

### SCTR expression correlated with liver fibrosis and DR

**Figure 3:**
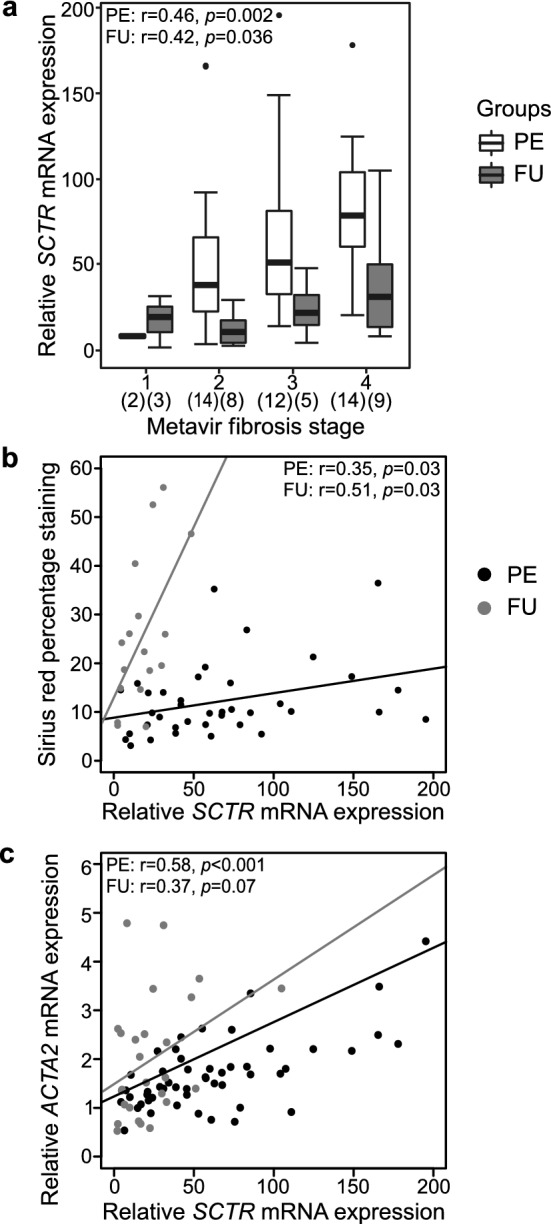
Correlations between liver secretin receptor (*SCTR*) mRNA expression and surrogates for liver fibrosis. (**a**) Box plot (median, interquartile range and 90th percentile) of relative *SCTR* expression in biliary atresia patients at portoenterostomy (PE) and during follow-up (FU) according to Metavir fibrosis stages. Correlation between *SCTR* and (**b**) Sirius red percentage staining and (**c**) *ACTA* mRNA expression. Black dots represent samples obtained at PE and grey dots represent follow-up (FU) samples after successful PE.

Next, we addressed the possible mechanisms underlying the unique prognostic potential of *SCTR* by correlating liver mRNA expression with histological, immunohistochemical and transcriptional surrogates for liver fibrosis and DR. *SCTR* expression positively correlated with Metavir fibrosis stage, proportional Sirius red staining area and liver mRNA expression of *ACTA*, encoding α-SMA, a marker for activated myofibroblasts, both at the time of PE and during the follow-up after SPE (Fig. 3). In addition, liver *SCTR* mRNA expression showed strong positive correlations with liver expression of *TGFB2* (r = 0.82, *P* < 0.001), *PDGFA* (r = 0.75, *P* < 0.001) and *PDGFB* (r = 0.63, *P* < 0.001) at the time of PE (Supplementary Fig. S6 available online).

**Figure 4:**
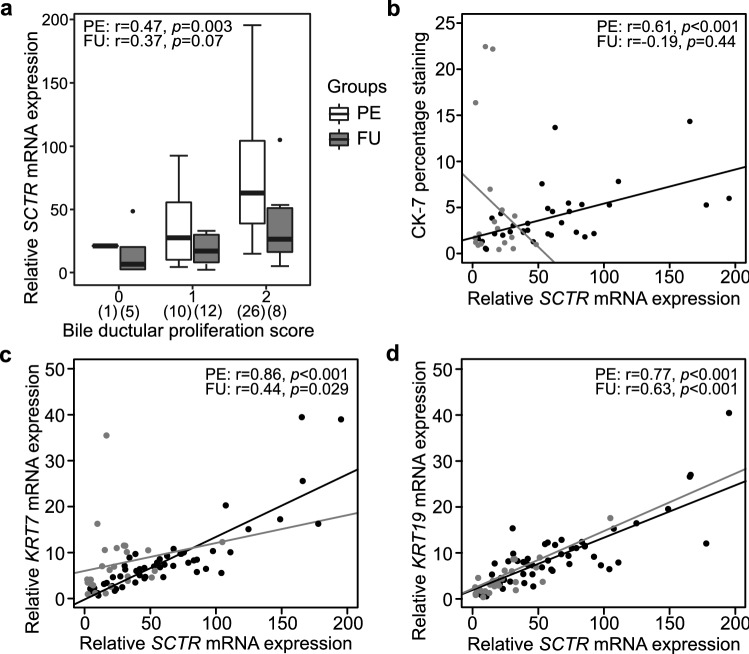
Correlations between liver secretin receptor (*SCTR*) mRNA expression and surrogates for ductular reaction. (**a**) Box plot (median, interquartile range and 90th percentile) of relative *SCTR* expression in biliary atresia patients at portoenterostomy (PE) and during follow-up (FU) according to CK-7 positive bile ductular score. Correlation between *SCTR* and (**b**) CK-7 percentage staining, (**c**) *KRT7* and (**d**) *KRT19* mRNA expression. Black dots represent samples obtained at PE and grey dots represent follow-up (FU) samples after successful PE.

At PE, *SCTR* showed strong positive associations with bile ductular proliferation score, proportional CK-7 staining area and mRNA expression of cholangiocyte markers *KRT7* and *KRT19* (Fig. 4). During the follow-up after SPE, the significant correlations with *KRT7* and *KRT19* remained, while the positive correlation with bile ductular proliferation failed to reach statistical significance. No correlation was found between *SCTR* and CK-7 staining area in follow-up biopsies (Fig. 4).

### SCTR was expressed by cholangiocytes in DR region

**Figure 5:**
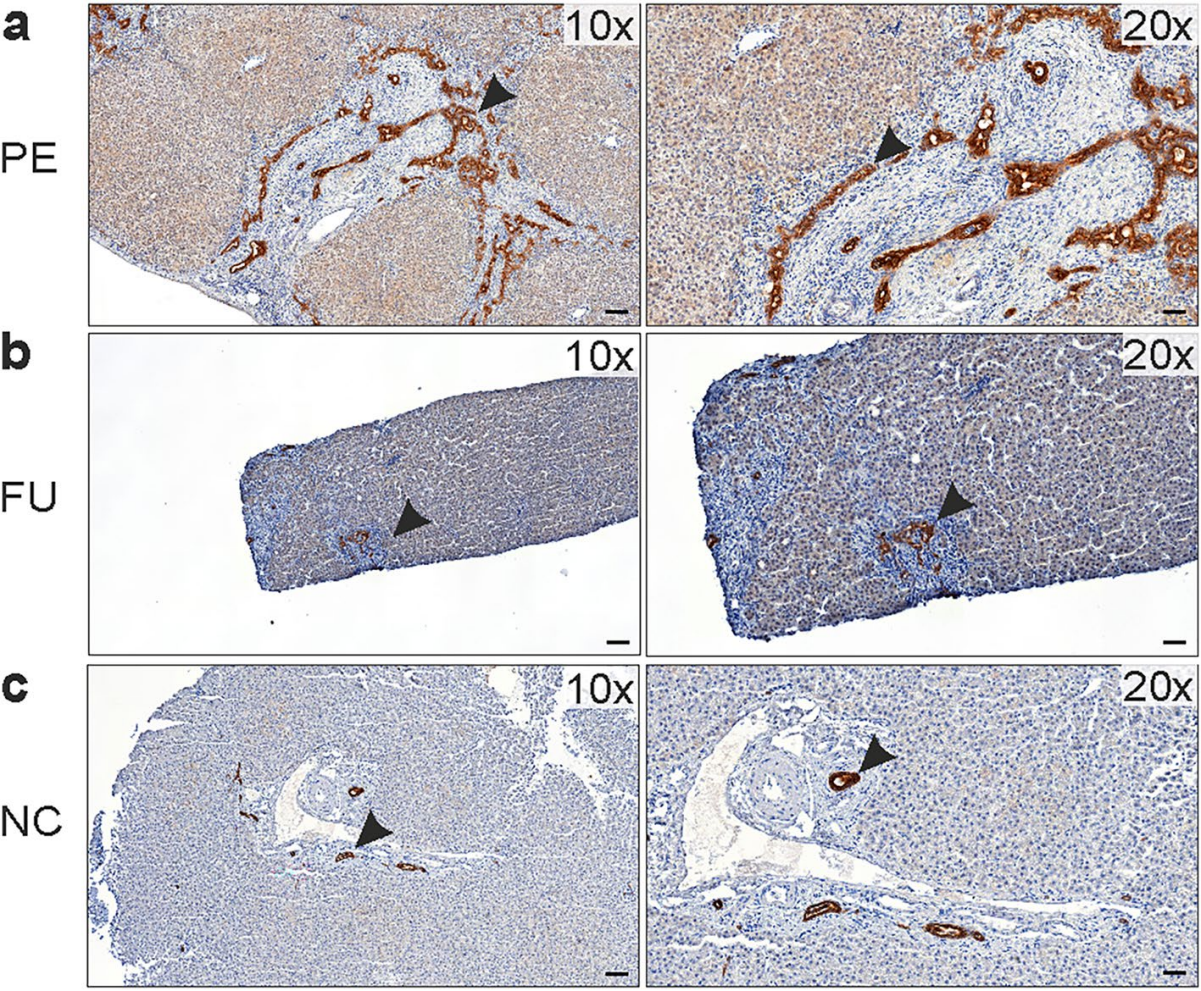
Representative immunohistochemistry on liver expression of secretin receptor (SCTR) in biliary atresia (**a**) at the time of portoenterostomy (PE) and (**b**) during follow-up (FU) 1.4 years after successful PE and (**c**) in a normal control patient (NC). Black arrow heads point to SCTR expressing cholangiocytes (brown). Note SCTR expression in all cholangiocytes including the developing neoductules in the ductular region of biliary atresia patients. Physiological SCTR expression is also seen in large bile duct cholangiocytes of normal liver.

On immunohistochemistry, distinct SCTR expression was present in the cholangiocytes of DR region bile ducts in BA samples obtained both at PE and after SPE (Fig. 5). This expression was seen uniformly in large and small cholangiocytes as well as in the developing neo-ductules in DR regions. SCTR expression was localized to the cell membranes of cholangiocytes predominantly in the basolateral regions, while no expression in hepatocytes was observed. Normal control patients showed physiological SCTR expression in large bile duct cholangiocytes.

### SCTR associated with PE age and postoperative AST levels

*SCTR* correlated positively with patient age at the time of PE (r = 0.47, *P* < 0.001, n = 53), and with AST (r = 0.43, *P* = 0.03, n = 25) during postoperative follow-up after SPE.

## Discussion

Herein, we have examined the prognostic and pathophysiological significance of 22 predetermined differentially expressed BA specific candidate genes^17^. Among them, SCTR showed unique associations with PE outcomes and liver injury. Our main new findings showed that *SCTR* is overexpressed by over 50-fold at the time of PE, when the high expression levels predicted decreased clearance of jaundice and 5-year NLS. Following SPE, *SCTR* expression decreased steadily along with increasing follow-up time after SPE eventually reaching down to the control levels after 8 years, demonstrating absence of aberrant *SCTR* expression among patients with extended NLS. Among the different liver cells, SCTR was localized to cholangiocytes and associated with liver fibrosis and DR both at the time of PE and after SPE.

SCTR is a G-protein coupled receptor protein binding to the neuroendocrine hormone secretin, which is mainly produced by the S-cells in the small intestine^18^. Secretin and SCTR have also been demonstrated in several other organs including cholangiocytes in the liver^19–21^. In the liver, the secretin/SCTR axis regulates bicarbonate rich secretion from cholangiocytes maintaining ‘bicarbonate umbrella’, which protects the bile duct epithelium against high bile acid concentrations^21^. Abolishing the secretin/SCTR axis in bile duct ligated SCTR^−^/^−^ knockout mice decreases cholangiocyte proliferation, expansion of DR and liver fibrosis^22,23^. Treatment with SCTR antagonists has been demonstrated to produce similar changes in mouse models of primary sclerosing cholangitis (PSC) and primary biliary cholangitis (PBC) by reducing TGF-β1 mediated activation of hepatic stellate cells, suggesting that secretin/SCTR has an important role in regulating DR driven liver fibrosis in cholangiopathies^23–25^. In line with the experimental findings, patients with PSC and PBC show increased liver expression of SCTR^24,25^. We extend these previous observations by demonstrating overexpression of *SCTR* in BA livers with a close correlation to liver fibrosis both at PE and after SPE. Collectively, these findings suggest that the secretin/SCTR axis is relevantly involved with DR and progression of liver injury also in BA.

Under physiological conditions, SCTR is expressed in large cholangiocytes unless biliary damage and consequent proliferation occurs, when also small neocholangiocytes acquire SCTR expression^18,21,26^. Accordingly, we observed immunohistochemical SCTR expression uniformly in all cholangiocytes of DR regions in BA. The presence of more active bile ductular proliferation at the time of PE contemplates the reducing *SCTR* expression trend in the long-term native liver survivors following SPE. Although *SCTR* expression was uniformly associated with ductular proliferation and transcriptional cholangiocyte markers CK-7 and CK-19, *SCTR* correlated with CK-7 positive area fraction only at the time of PE. The lacking correlation observed during follow-up most likely results from increasingly acquired CK-7 expression also by transdifferentiating periportal hepatocytes, not expressing SCTR, after SPE^27,28^. Nevertheless, our findings support and may provide an explanation for previous findings connecting a high rate CK-7 positive biliary proliferation at PE with advanced liver fibrosis, poor bile drainage and decreased NLS^29,30^.

Our study showed *SCTR* to have a distinct prognostic value with regards to clearance of jaundice and NLS. The patients with the highest expression had the worst prognosis. Meanwhile, *SCTR* showed consistent positive correlations with different histological, immunohistochemical and transcriptional surrogates of liver fibrosis, providing a logical explanation for the observed prognostic potential. The association between *SCTR* and liver fibrosis was further strengthened by close correlations of *SCTR* with TGF-β and *PDGF*, growth factors known to mediate liver fibrosis^31,32^. However, unlike in previous experimental studies, *SCTR* associated with *TGFB2* rather than *TGFB1*, which is less abundantly expressed TGF-β isoform in BA^17,33,34^. Additional studies in cholestatic experimental animal models are needed to clarify whether the secretin/SCTR axis could be targeted to reduce fibrosis and biliary damage in BA^20,22,25^.

LAMC2, an extracellular matrix-related basement membrane protein, has been linked as a prognosticator for several cancers^35,36^. As acquisition of apical-basal polarity and orientation is elemental for proliferative cholangiocytes in DR^12^, the aberrant expression of *LAMC2* documented in BA may be related to its important physiological role in cell adhesion^37,38^. Interestingly, disrupted apical-basal localization of SCTR was recently reported in BA organoids^39^.

Our study had some limitations and strengths. The main limitations include the retrospective study design, which invariably leads to imperfections in data retrieval and intervals, while providing no proof of causality. Only few patients had samples obtained both at PE and during follow-up, precluding our ability to address longitudinal changes in individual patients. Due to lack of samples, we were not able to address prognostic value of serum secretin levels, which would be a logical next step. Our strengths include a relatively large study cohort with unique sample collection covering a long postoperative period. The candidate genes studies were predetermined, and the patients were compared to carefully selected normal and disease controls of appropriate ages.

In conclusion, we demonstrate significant liver overexpression of SCTR, which predicted clearance of jaundice and NLS in BA and correlated closely with liver fibrosis both at the time of PE and during postoperative follow-up after SPE.

## Methods

### Patients and controls

We included all patients with archived liver biopsies, who underwent PE surgery between 2005–2012 in King’s College Hospital, London, UK and between 2012–2020 in the Children’s Hospital, University of Helsinki, Finland. Overall, liver biopsies were obtained at PE from 50 patients, during postoperative follow-up after SPE from 22 patients, and at both study points from 3 patients (Table 1). As post-PE liver biopsies are not standard clinical management in King’s College Hospital in London, all follow-up biopsies were collected from stable patients in Helsinki^27^. Wedge liver biopsies were obtained at PE and ultrasound guided core needle biopsies under general anesthesia for endoscopic variceal surveillance during follow-up as described previously^27,40^. The BA diagnosis was confirmed by histopathological assessment of liver biopsy and bile duct remnant in all cases. All PE surgeries were open, and steroids, ursodeoxycholic acid (UDCA) and antibiotics were routinely used postoperatively in both centers.

As normal controls (NC), we included 20 liver samples obtained at median (IQR) age 6.5 (1.1–25) years, including 14 donor liver biopsies and 6 commercially available pediatric liver biopsy homogenates (H0260, H0268, H0551, H0845, H0872 and H1393, Sekisui XenoTech, Kansas City, KS, USA). As disease controls, we included 22 diagnostic liver biopsies from children with other pediatric liver disorders, who were further divided into two groups. Cholestatic young disease controls (YDC), with median age of 122 (41–203) days (*P* = 0.063 versus patients at PE), were compared against cholestatic BA patients at PE. All old disease controls (ODC) with median age of 9.2 (6.0–10.9) years were operated for choledochal malformations. ODC patients were not cholestatic at the time of liver biopsy obtained during choledochal malformation surgery, as they were compared against non-cholestatic BA study patients during the follow-up period after SPE (Table 1). Clinical details of disease control patients are shown in Supplementary Table S1 available online.

### Liver mRNA expression

RNA was extracted from liver biopsy samples following the manufacturer’s instructions using RNeasy Mini Kit (QIAGEN, Frederick, MD, USA). MRNA expression of the 22 ECM molecular fingerprint genes (*COL10A1*, *COL15A1*, *COL22A1*, *COL9A2*, *CTHRC1*, *ENTPD2*, *ITGB6*, *LAMA3*, *LAMC2*, *MMP7*, *VWA2*, *WNT10A*, *CKAP2L*, *CLDN19*, *FAP*, *HMMR*, *KRT222*, *MROH7*, *NCAPG*, *SCTR*, *STMN2* and *TMEM178B*) was analyzed by quantitative real-time polymerase chain reaction (qPCR) using Custom RT Profiler PCR Array (QIAGEN) on BIO-RAD CFX384 Real-Time System (Bio-Rad, Hercules, CA, USA) according to the manufacturer’s instructions. In addition, we analyzed mRNA expression of *ACTA2*, encoding α-SMA, a marker of myofibroblast activation, and *KRT7* and *KRT19,* encoding cholangiocyte markers cytokeratin-7 (CK-7) and CK-19 as well as two growth factors essentially involved with liver fibrogenesis: transforming growth factor beta (*TGFB1*, *TGFB2*) and platelet derived growth factor (*PDGFA*, *PDGFB*). Quantification was performed using the ∆∆Ct method after normalization to housekeeping genes *HPRT1*, *GAPDH*, *ACTB* and *B2M* and relative to normal control patients.

### Liver biochemistry and histology

Serum samples were analyzed in the local hospital laboratory for bilirubin, alanine aminotransferase (ALT), aspartate transaminase (AST), and gamma-glutamyl transferase (GGT). AST-to-platelet ratio index (APRi) was calculated^41^. Two experienced pediatric pathologist, who were blinded to the clinical data, graded the biopsy samples for fibrosis using the Metavir fibrosis staging (0–4) and CK-7 positive bile ductular proliferation (0–2) as described previously^27^.

### Immunohistochemistry and image analyses

Formalin fixed paraffin embedded sections were deparaffinized, hydrated, and treated with target retrieval solution at pH 6 (Dako—Agilent Technologies, Glostrup, Denmark). Commercially available antibody for SCTR (rabbit polyclonal, ab224236, Abcam, Cambridge, UK) was used at a dilution of 1:1000 along with the Novolink TM polymer detection system (Leica biosystems Newcastle Ltd, Newcastle Upon Tyne, UK). CK-7 staining was performed at the Diagnostic Center of Hospital District of Helsinki and Uusimaa, using primary antibody (790–4462, Ventana, Roche, Basel, Switzerland) in a fully automated Ventana Benchmark Ultra IHC system^27^. Collagen fibers were stained with Sirius red (ab150681, Abcam, Cambridge, UK) following the manufacturer’s instructions. Proportional CK-7 and Sirius red positive areas were defined and measured by colour intensities and shade using CaseViewer HistoQuant Software (3DHISTECH, Budapest, Hungary). The parenchymal area of the biopsy section was utilized, avoiding the liver capsule, and used for the software analyses. All images were generated using 3DHISTECH Panoramic 250 FLASH II digital slide scanner at Genome biology unit supported by HiLIFE and the Faculty of Medicine, University of Helsinki, and Biocenter Finland.

### Statistics

All continuous variables are expressed as median and interquartile range (IQR). Comparisons between two groups were performed using the Mann–Whitney U test and multiple group comparisons with the Kruskal–Wallis test. Correlations were tested using the Spearman’s rank correlation. To examine the effects of different mRNA expression levels on NLS, Kaplan–Meier analysis with log-rank test of expression level tertiles for each gene of interest was performed. *P* < 0.05 was considered statistically significant and all analyses were done on RStudio version 1.3.1093 (RStudio, Boston, MA, USA).

### Ethics

The study protocol was approved by ethical committees at both participating hospitals. The study was approved by the hospital ethical committee (protocol number 345/03/1,372,008) and the institutional review board on 21 July 2017 (§68 HUS/149/2017) in Finland and by the National Research Ethics Committee (12/WA/0282 and 18/SC/0058) in the UK. All research methods were performed in accordance with the relevant guidelines and regulations. Informed consent for use of samples in research was obtained from all patients/legal guardians.

## Supplementary Information

Supplementary Information.

## Data Availability

The datasets generated and/or analyzed during the current study are not publicly available as it contains unpublished data we are working on, but are available from the corresponding author on reasonable request.

## Abbreviations

ALT: Alanine aminotransferase
α-SMA: Alpha smooth muscle actin
APRi: AST-to-platelet ratio index
AST: Aspartate transaminase
BA: Biliary atresia
CI: Confidence interval
CK: Cytokeratin
DR: Ductular reaction
ECM: Extracellular matrix
GGT: Gamma glutamyl transferase
LAMC2: Laminin subunit gamma-2
LT: Liver transplantation
mRNA: Messenger RNA
NC: Normal control
NLS: Native liver survival
ODC: Old disease control
PBC: Primary biliary cholangitis
PDGF: Platelet derived growth factor
PE: Portoenterostomy
PSC: Primary sclerosing cholangitis
qPCR: Quantitative polymerase chain reaction
SCTR: Secretin receptor
SPE: Successful portoenterostomy
TGFB: Transforming growth factor beta
YDC: Young disease control
