# Supplementary material for: Liver secretin receptor predicts portoenterostomy outcomes and liver injury in biliary atresia
Source: Sci Rep. 2022 May 4;12:7233. doi: 10.1038/s41598-022-11140-9 (PMC9068784; doi:10.1038/s41598-022-11140-9)
Supplement: Supplementary file 1 — Supplementary Information. [file 41598_2022_11140_MOESM1_ESM.pdf]

# Liver secretin receptor predicts portoenterostomy outcomes and liver injury in biliary atresia

Nimish Godbole[1,2], Iiris Nyholm[1,2], Maria Hukkinen[1,2], Joseph R Davidson[3,4], Athanasios Tyraskis[4], Jouko Lohi[5], Päivi Heikkilä[5], Katja Eloranta[1], Marjut Pihlajoki[1], Mark Davenport[4], Markku Heikinheimo[1,6], Antti Kyrönlahti[1], Mikko P Pakarinen[1,2*]

[1] Pediatric Research Center, Children's Hospital, University of Helsinki and Helsinki University Hospital, Helsinki, Finland

[2] Section of Pediatric Surgery, Pediatric Liver and Gut Research Group and Pediatric Research Center, Children's Hospital, University of Helsinki and Helsinki University Hospital, Helsinki, Finland

[3] Department of Pediatric Surgery, GOS-UCL Institute of Child Health, London, United Kingdom

[4] Department of Pediatric Surgery, King's College Hospital, London, United Kingdom

[5] Department of Pathology, University of Helsinki and Helsinki University Hospital, Helsinki, Finland

[6] Department of Pediatrics, Washington University in St. Louis, MO, USA

* Corresponding author: Mikko P. Pakarinen, MD, PhD, Helsinki University Hospital, Children's Hospital, P.O. Box 281, 00029 HUS, Helsinki, Finland

E-mail: mikko.pakarinen@hus.fi

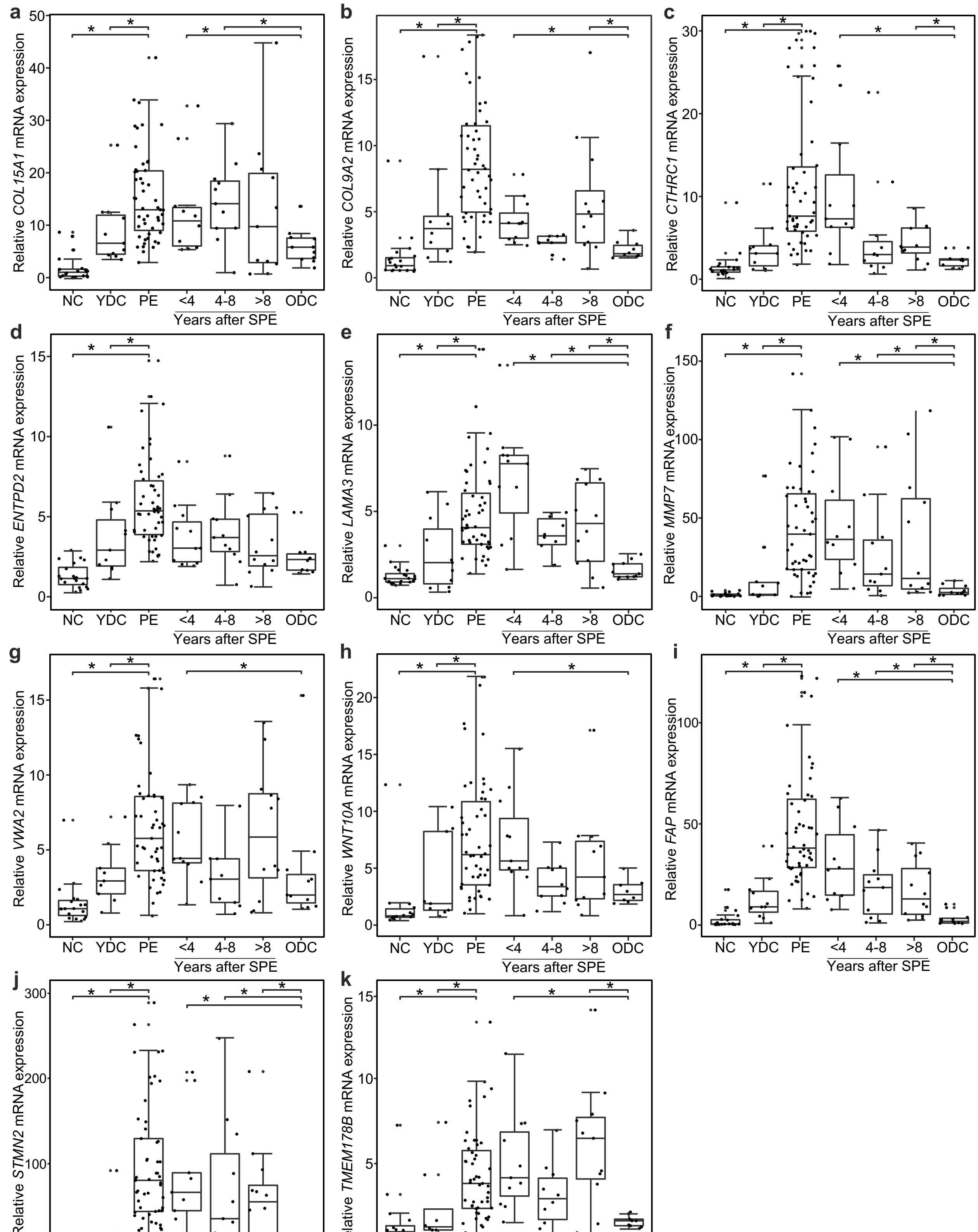


**Supplementary figure S1:** Relative expression of biliary atresia specific genes (in addition to *SCTR* and *LAMC2*) significantly overexpressed at portoenterostomy (PE) compared to normal controls (NC) and young disease controls (YDC). Box plot (median, interquartile range and 90$^{th}$ percentile with jittered data points) of (a) *COL15A1* (b) *COL9A2* (c) *CTHRC1* (d) *ENTPD2* (e) *LAMA3* (f) *MMP7* (g) *VWA2* (h) *WNT10A* (i) *FAP* (j) *STMN2* and (k) *TMEM178B* in NC (n=20), YDC (n=11), old disease controls (ODC, n=11), biliary atresia patients at PE (n=53) and during follow-up after successful PE (SPE; <4 years n=11, 4-8 years n=11, >8 years n=12) *p<0.05

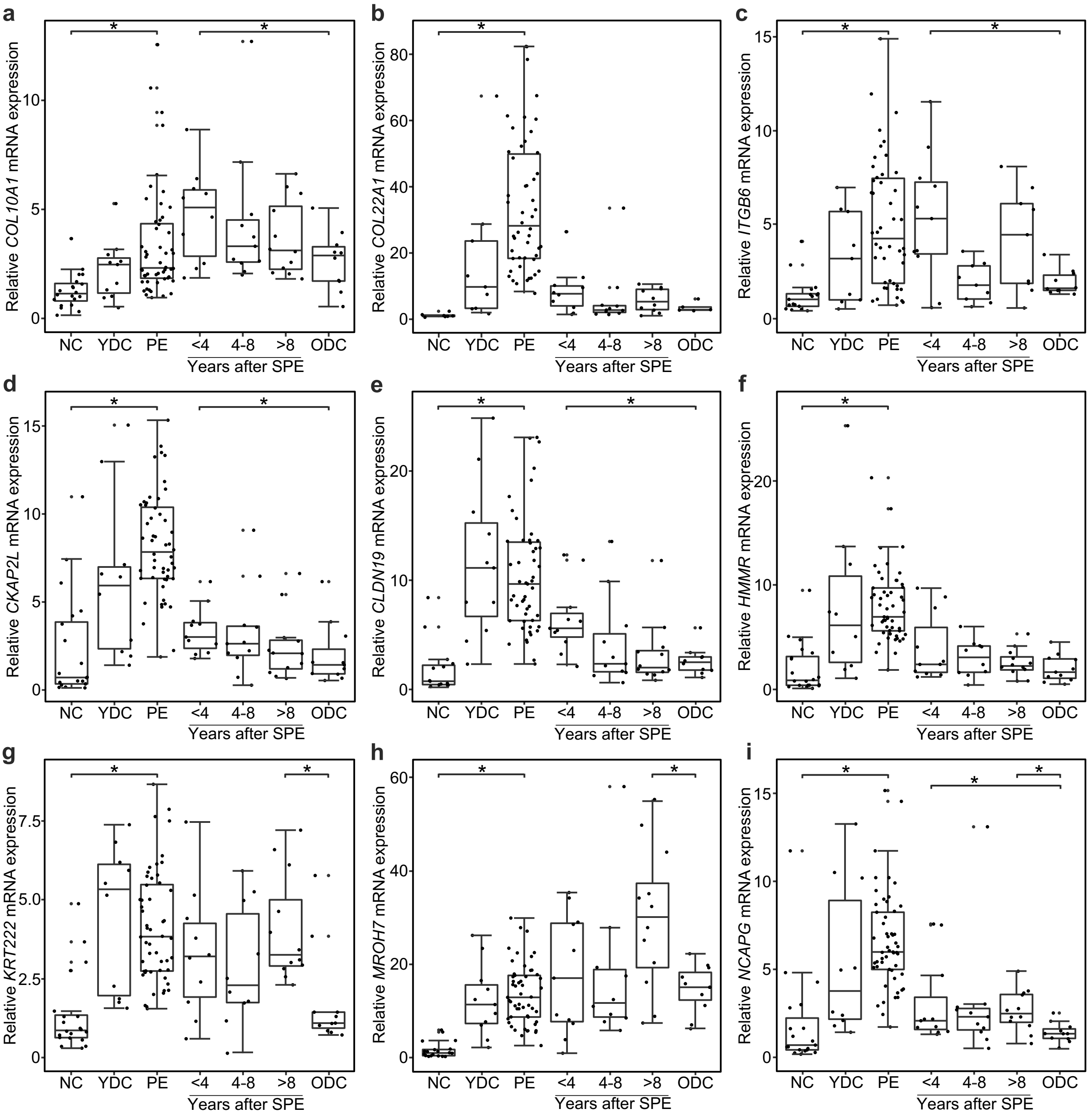


**Supplementary figure S2:** Relative expression of genes significantly overexpressed at portoenterostomy (PE) compared to normal controls (NC) but not young disease controls (YDC). Box plot (median, interquartile range and 90th percentile with jittered data points) of (a) *COL10A1* (b) *COL22A1* (c) *ITGB6* (d) *CKAP2L* (e) *CLDN19* (f) *HMMR* (g) *KRT222* (h) *MROH7* and (i) *NCAPG* in NC (n=20), YDC (n=11), old disease controls (ODC, n=11), biliary atresia patients at PE (n=53) and during follow-up after successful PE (SPE; <4 years n=11, 4-8 years n=11, >8 years n=12) *$p<0.05$

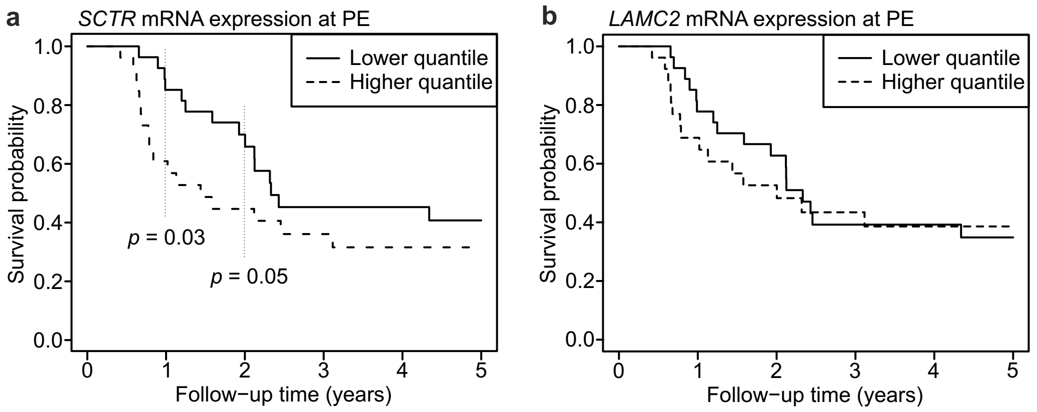


**Supplementary figure S3:** Portoenterostomy (PE) outcomes according to secretin receptor (*SCTR*) and laminin subunit gamma-2 (*LAMC2*) expression. Kaplan-Meier survival curves for native liver survival according to quantile of mRNA liver expression in (a) *SCTR* and (b) *LAMC2* at PE (n=53). Significant p-values are shown separately for each follow-up year

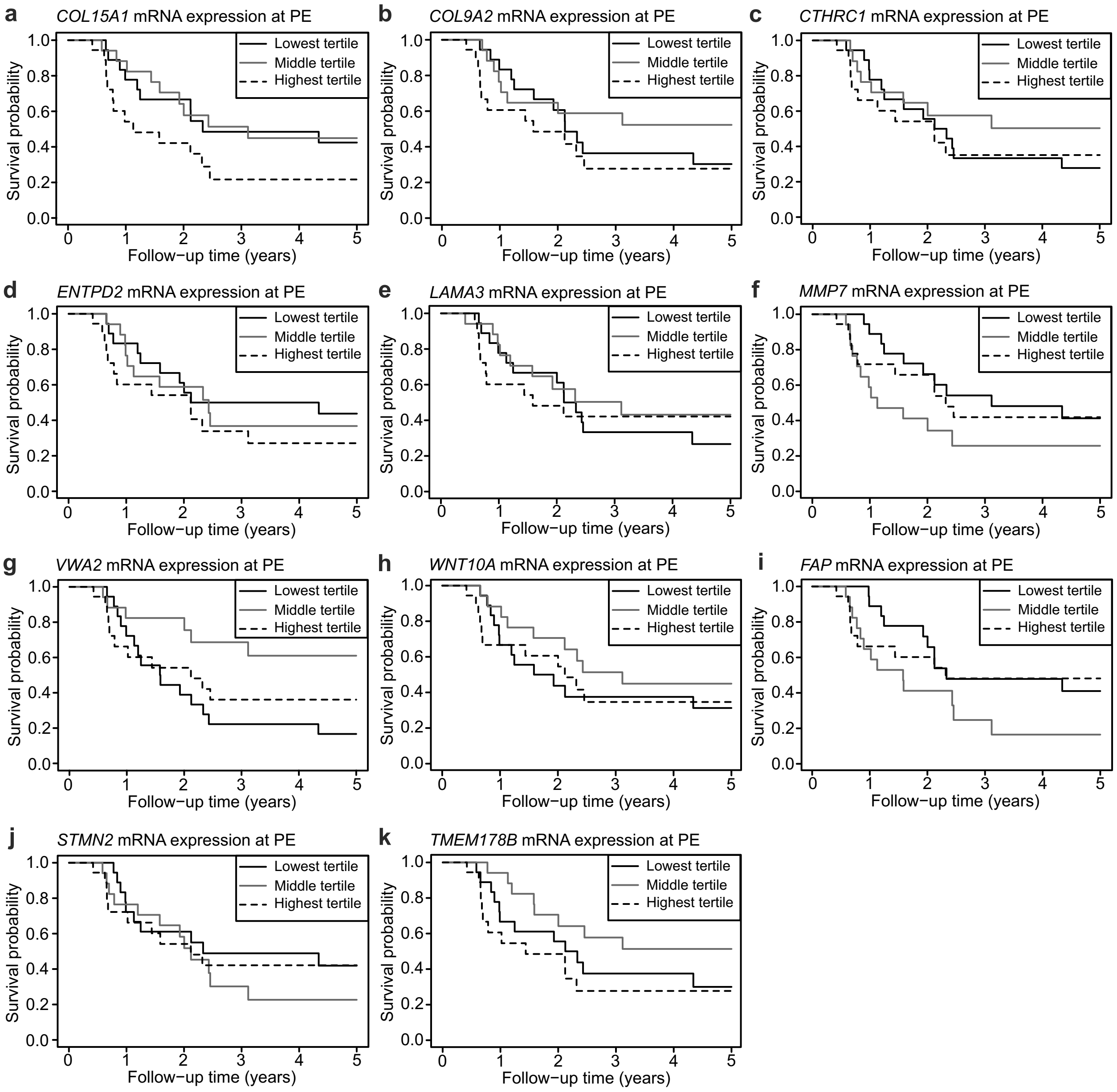


**Supplementary figure S4:** Kaplan Meier curves for native liver survival according to mRNA liver expression tertiles at portoenterostomy (PE, n=53) for the genes that were significantly overexpressed at PE compared to normal and (young) disease controls. (a) *COL15A1* (b) *COL*9A2 (c) *CTHRC1* (d) *ENTPD*2 (e) *LAMA3* (f) *MMP7* (g) *VWA2* (h) *WNT10A* (i) *FAP* (j) *STMN2* and (k) *TMEM178B*. No significant differences between expression tertiles were observed

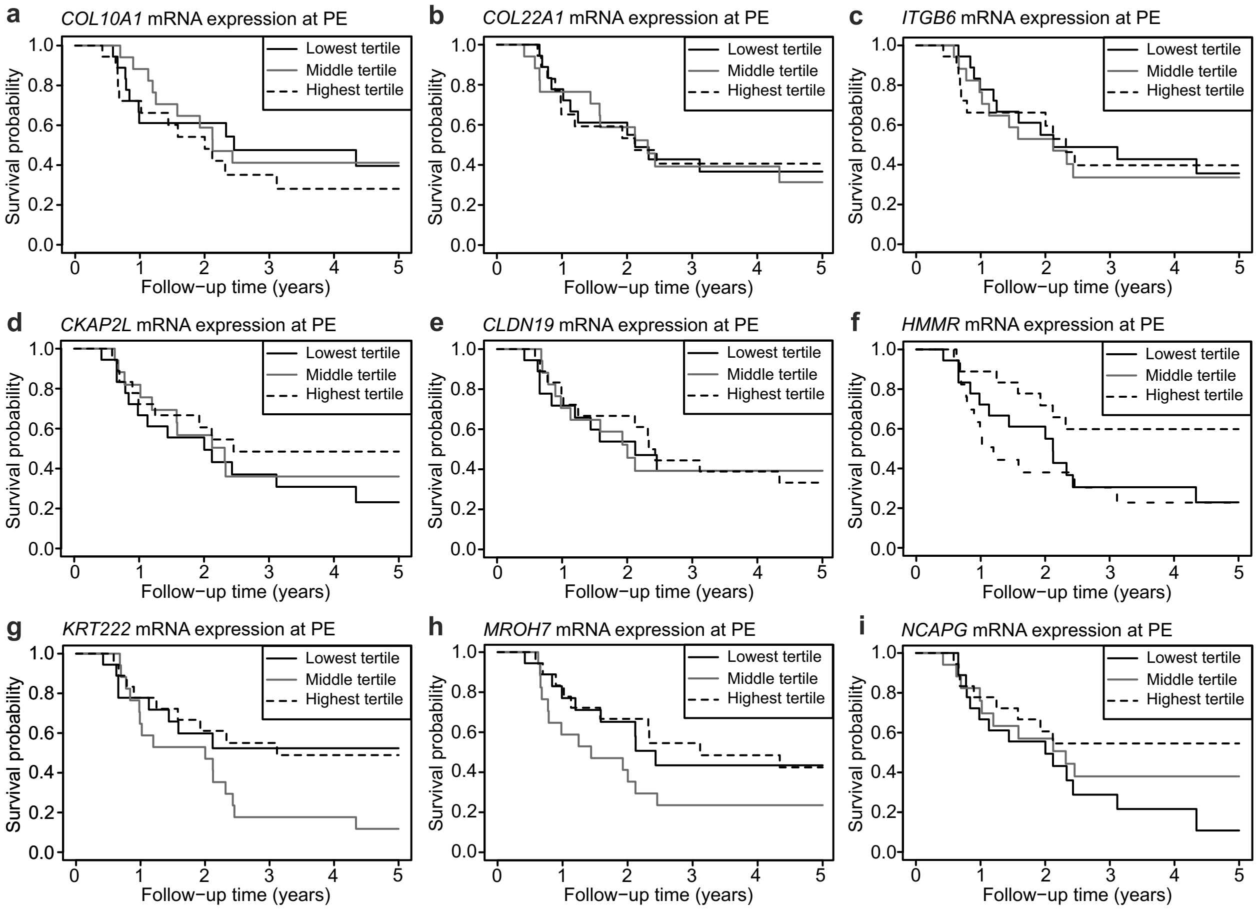


**Supplementary figure S5:** Kaplan Meier curves for native liver survival according to tertiles of mRNA liver expression at PE (n=53) for the genes that were significantly overexpressed at portoenterostomy in relation to normal but not (young) disease controls. (a) *COL10A1* (b) *COL22A1* (c) *ITGB6* (d) *CKAP2L* (e) *CLDN19* (f) *HMMR* (g) *KRT222* (h) *MROH7* and (i) *NCAPG*. No significant differences between expression tertiles were observed.

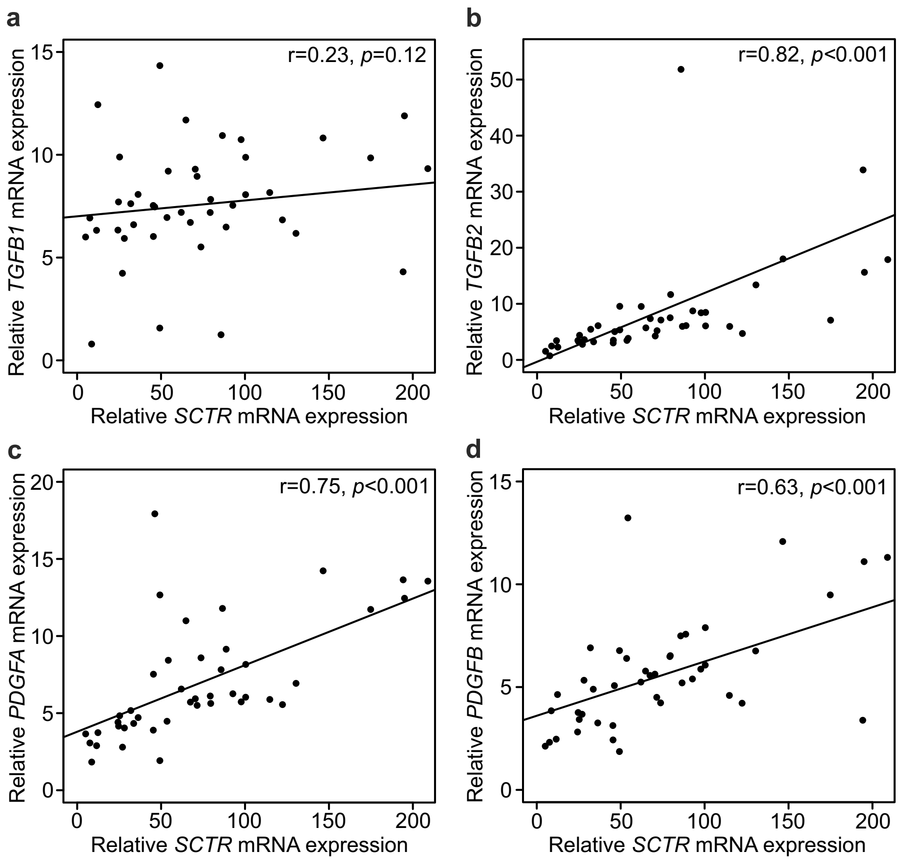


**Supplementary figure S6:** Correlation between liver mRNA expression of secretin receptor (*SCTR*) and (a) *TGFB1* (b) *TGFB2* (c) *PDGFA* and (d) *PDGFB* at the time of portoenterostomy

**Supplementary Table S1:** Baseline characteristics of disease controls.

| | Young disease controls | Old Disease controls |
|---|---|---|
| Controls, n | 11 | 11 |
| Female, n (%) | 7 (64) | 8 (73) |
| Age at liver biopsy, y | 0.33 (0.11 – 0.56) | 9.18 (5.95 – 10.85) |
| | | |
| Liver biochemistry | | |
| Bilirubin, µmol/l | 98 (42 – 129) | 10 (7 – 15) |
| Conjugated bilirubin, µmol/l | 83 (68 – 99) | 5 (3 – 7) |
| AST, U/l | 138 (68 – 153) | 78 (63 – 100) |
| ALT, U/l | 68 (49 – 111) | 76 (40 – 123) |
| GGT, U/l | 124 (90 – 292) | 19 (14 – 52) |
| APRi | 0.62 (0.41 – 1.10) | 0.56 (0.47 – 0.93) |
| | | |
| Diagnosis | | |
| Choledochal malformation, n | 5 | 11 |
| Biliary hypoplasia, n | 3 | 0 |
| α1-Antitrypsin deficiency, n | 2 | 0 |
| Undetermined cholestasis, n | 1 | 0 |

Data are median (IQR) or frequencies (%).
